# Decline in Stroke Mortality Between 1997 and 2012 by Sex: Ecological Study in Brazilians Aged 15 to 49 Years

**DOI:** 10.1038/s41598-019-39566-8

**Published:** 2019-02-27

**Authors:** Laércio da Silva Paiva, Fernando Rocha Oliveira, Luiz Vinicius de Alcantara Sousa, Francisco Winter dos Santos Figueiredo, Thiago Hérick de Sá, Fernando Adami

**Affiliations:** 1 Faculdade de Medicina do ABC. Laboratório de Epidemiologia e Análise de dados, Departamento de Saúde da Coletividade. Av. Lauro Gomes, 2000, Vila Sacadura Cabral, postcode: 09060-870 Santo André, SP Brazil; 2 Faculdade de Saúde Pública da Universidade de São Paulo, Departamento de Epidemiologia. Av. Dr. Arnaldo, 715, postcode: 01246-904 São Paulo, SP Brazil; 3 Universidade de São Paulo. Departamento de Nutrição da Faculdade de Saúde Pública. Av. Dr. Arnaldo, 715, postcode: 01246-904 São Paulo, SP Brazil

## Abstract

This study aimed to analyse the time trends of stroke mortality between 1997 and 2012 according to sex in Brazilians aged 15 to 49 years. This ecological study used data obtained from the Mortality Information System, which is available from the National Health System Department of Informatics - DATASUS and maintained by the Brazilian Ministry of Health. Stroke definition included International Classification of Disease version 10 (ICD-10) codes I60, I61, I63, and I64. Crude and age-standardized mortality rates and respective 95% confidence intervals were estimated per 100,000 inhabitants and stratified by age, region, year, and sex. Linear regression models were used to analyse the time trends with a confidence level of 95%. The statistical program used was Stata 11.0. Between 1997 and 2012, there were 124,866 deaths due to stroke in Brazilians aged 15 to 49 years. There was a decreasing linear trend in stroke mortality among men (β = −0.46, p < 0.001, R^2^ = 0.95) and women (β = −0.40, p < 0.001, R^2^ = 0.98) during this period. Overall there was no significant difference in stroke mortality trends by sex, except with respect to the age group of 40 to 49 years where there was a difference in the decrease of stroke mortality between men and women (interaction sex * year: β = 0.238, p = 0.012, R² = 0.96). Mortality rates decrease significantly over time in men and women in the age group 15 to 49 years old, but there is only significant difference in the decrease of rates by sex only in the age group from 40 to 49 years old.

## Introduction

In the period between 1980 and 2009, the incidence of stroke increased worldwide, particularly among adults younger than 50 years old^1–3^, owing to the increase in the prevalence of modifiable risk factors such as hypertension, dyslipidaemia, and diabetes^2,4,5^. Stroke is related to notably high morbidity and mortality rates in developed and developing countries^1^. Their consequences severely affect quality of life and work capacity, particularly among young adults^4,6^. Stroke events among individuals aged 15–49 years have a disproportionate impact on public health and work productivity owing to the loss of the most productive years of life^7^.

Stroke is the second leading global cause of death after ischaemic heart disease^2^, and in Brazil, it is a main cause of death, with more than 33,000 deaths reported in 2010^3^. However, advances in diagnostic methods, particularly neuroimaging, have improved the efficacy of medications, and patient care may have influenced the global reduction in stroke mortality^5,8,9^. Data from the Institute for Health Metrics and Evaluation^10^ indicate that global haemorrhagic stroke mortality rates (per 100,000 individuals) decreased in young adults from 10.7 (95% CI: 9.5; 12.1) in 1990 to 8.6 (95% CI: 7.6; 9.8) in 2013, whereas ischaemic stroke mortality rates remained stable during this period with rates of 1.8 (95% CI: 1.4; 2.3) in 1990 and 1.6 (95% CI: 1.3; 2.0) in 2013. When stratifying by sex, the same data suggest a reduction for both sexes, this reduction was greater in women with a higher mortality rate in men. In Brazil, there was a reduction in mortality from all stroke types among young adults from 7.5 (95% CI: 7.5; 7.5) in 2008 to 6.3 (95% CI: 6.3; 6.3) in 2012^11^; however, it is unclear whether this trend differs between sexes^12–15^.

Differences between women and men exist regarding immunity; coagulation; hormonal factors; reproductive factors, including pregnancy and childbirth; and social factors, which may influence the risk for stroke and consequently mortality over the years^16^. Therefore, monitoring risk factors in this population for each sex, creating policies aimed at primary prevention, improving health care, and advancing research related to treatment and stroke rehabilitation are essential, because in the long term, it may change the scenario of stroke mortality in this population. Thus, our objective was to analyse the sex-specific time trends of stroke mortality between 1997 and 2012 among Brazilians aged 15 to 49 years.

## Methods

### Study design, data source and data collection

This ecological study was conducted in September 2015 and included cases of deaths reported as stroke-related in Brazilians between 15 and 49 years of age, who were defined as individuals within this age group who were living in the Brazilian territory between January 1, 1997 and December 31, 2012.

Data for stroke mortality was extracted from the Mortality Information System (*Sistema de Informação sobre Mortalidade* - SIM) available on the website of the Department of Informatics of the National Health System (DATASUS) (http://www2.datasus.gov.br/). The SIM receives, processes, verifies, validates, and makes available data on deaths occurring in Brazil. In 2011, the SIM covered 96.1% of all deaths in the country^17^, although coverage is lower in the North (83.1%) and Northeast (87.1%) regions of the country^18^.

We used the following International Classification of Diseases version 10 (ICD-10) codes on DATASUS: subarachnoid haemorrhage (I60), intracranial haemorrhage (I61), cerebral infarction (I63), and stroke not specified as ischaemic or haemorrhagic (I64)^19,20^. We obtained data related to sex (male and female), age range (15–19, 20–24, 25–29, 30–34, 40–44, 45–49 years), administrative Brazilian regions (North, Northeast, Southeast, South and Center-West), stroke subtypes (i.e. subarachnoid haemorrhagic, intracerebral haemorrhagic, ischaemic, and non-specified) and number of deaths per year (1997 to 2012). In addition, the 2000–2010 total population census and projections weres used in mortality ratios analysis^21^. These data are provided by the Brazilian Institute of Geography and Statistics (Instituto Brasileiro de Geografia e Estatística - IBGE), which conducts a census every 10 years to verify the Brazilian population profile, and estimates by projections the population size in the years between censuses.

All DATASUS data compilation was performed by two researchers independently; a third researcher was responsible for extracting additional data and correcting discrepancies.

### Statistical analysis

Non-standardized stroke mortality was estimated for both sexes by calculating the number of stroke deaths in each age group by country region and year divided by the total population at risk in that age group, administrative region, and year and multiplied by 100,000 inhabitants. Sex-stratified stroke mortality was standardized by age (direct method), with the standard population of the World Health Organization^22^ used as the reference population. Proportional mortality by groups of causes was calculated by dividing the number of deaths per group of defined causes by the total number of deaths, excluding ill-defined causes (ICD-10, R00-R99)^21^.

Deaths from stroke were described using absolute frequency, relative frequency, and proportional mortality. The 95% confidence interval of the proportion was based on the calculation, upper limit (Sample Proportion + 1.96 × Standard Error) and lower limit (Sample Proportion − 1.96 × Standard Error)^23,24^.

A linear regression was used to analyse the time trends of stroke mortality between 1997 and 2012, stratified by sex and age group. For this analysis the slope (β), the probability value (p) and the model fit (R^2^) were extracted from the linear regression. The regression model used was as follows: y = β0 + β1 * t, in which y = mortality by stroke (per 100,000 inhabitants), t = calendar years, β0 = intercept, and β1 = slope, which corresponds to the mean annual change in stroke mortality.

To analyse the difference between sex over time, we included in the regression analysis the interaction model: y = β0 + β1 * sex + β2 * t + β3 * sex * t, where y = mortality by stroke (per 100,000 inhabitants), t = calendar years, β0 = intercept, β1 = slope, β2 = slope, and β3 corresponds to the interaction between sex and year. Extracted from the regression interaction model were the slope (β), the probability value (p) and the model fit (R^2^).

Data analyses were conducted with Stata (Data Analysis and Statistical Software) version 11.0.

## Results

Between 1997 and 2012, 124,866 deaths from stroke have been reported in individuals aged 15 to 49 years residing in Brazil. Of those deaths, 62,751 occurred in men, and 62,115 occurred in women. In both sexes, deaths from stroke occurred in older age groups, mainly after 35 years of age; 82.8% and 83.5% of deaths occurred in men and women of this age group, respectively (Table 1).

**Table 1:** Frequency of deaths from stroke* in Brazilian men and women aged 15 to 49 years between 1997 and 2012.

Characteristics	Deaths			
	Men		Women	
	N	% (95% CI)	N	% (95% CI)
**Age group (years)**				
15|–20	1,206	1.9 (1.1; 2.7)	1,027	1.7 (0.9; 2.5)
20|–25	1,851	2.9 (2.1; 3.7)	1,651	2.7 (1.9; 3.5)
25|–30	2,779	4.4 (3.6; 5.2)	2,658	4.3 (3.5; 5.1)
30|–35	4,926	7.9 (7.1; 8.7)	4,906	7.9 (7.1; 8.7)
35|–40	9,381	14.9 (14.2; 15.6)	9,777	15.7 (15.0; 16.4)
40|–45	16,696	26.6 (25.9; 27.3)	17,455	28.1 (27.4; 28.8)
45|–50	25,912	41.3 (40.7; 41.9)	24,641	39.7 (39.1; 40.3)
**Regions**				
North	3,663	5.8 (5.0; 6.6)	3,520	5.7 (4.9; 6.5)
Northeast	14,025	22.4 (22.7; 23.1)	14,763	23.8 (23.1; 24.5)
Southeast	32,128	51.2 (50.7; 51.7)	30,931	49.8 (49.2; 50.4)
South	8,624	13.7 (13.0; 14.4)	8,609	13.9 (13.2; 14.6)
Midwest	4,311	6.9 (6.1; 7.7)	4,292	6.9 (6.1; 7.7)
**Stroke subtypes**				
Subarachnoid haemorrhagic (I60)	8,683	13.8 (13.1; 14.5)	15,559	25.0 (24.3; 25.7)
Intracerebral haemorrhagic (I61)	26,809	42.7 (42.1; 43.3)	22,579	36.4 (35.8; 37.0)
Ischaemic (I63)	2,259	3.6 (2.8; 4.4)	2,038	3.3 (2.5; 4.1)
Non-specified (I64)^¥^	25,000	39.8 (39.2; 40.4)	21,939	35.3 (34.7; 35.9)
**Calendar year**				
1997	4,707	7.5 (6.7; 8.3)	4,437	7.1 (6.3; 7.9)
1998	4,769	7.6 (6.8; 8.4)	4,469	7.2 (6.4; 8.0)
1999	4,557	7.3 (6.5; 8.1)	4,423	7.1 (6.3; 7.9)
2000	4,223	6.7 (5.9; 7.5)	4,335	7.0 (6.2; 7.8)
2001	4,242	6.8 (6.0; 7.6)	4,147	6.7 (5.9; 7.5)
2002	4,000	6.4 (5.6; 7.2)	4,010	6.5 (5.7; 7.3)
2003	4,011	6.4 (5.6; 7.2)	4,114	6.6 (5.8; 7.4)
2004	3,991	6.4 (5.6; 7.2)	3,984	6.4 (5.6; 7.2)
2005	3,662	5.8 (5.0; 6.6)	3,769	6.1 (5.3; 6.9)
2006	3,830	6.1 (5.3; 6.9)	3,706	6.0 (5.2; 6.8)
2007	3,598	5.7 (4.9; 6.5)	3,673	5.9 (5.1; 6.7)
2008	3,630	5.8 (5.0; 6.6)	3,705	6.0 (5.2; 6.8)
2009	3,416	5.4 (4.6; 6.2)	3,432	5.5 (4.7; 6.3)
2010	3,425	5.5 (4.7; 6.3)	3,350	5.4 (4.6; 6.2)
2011	3,370	5.4 (4.6; 6.2)	3,390	5.5 (4.7; 6.3)
2012	3,320	5.3 (4.5; 7.0)	3,171	5.1 (4.3; 5.9)
Total	62,751	100.0	62,115	100.0

95% CI: 95% confidence interval.

*International Statistical Classification of Diseases and Related Health Problems–10th revision (ICD-10) codes: I60, I61, I63 and I64^19,20^.

^¥^Stroke not specified as ischaemic or haemorrhagic.

Data source: Mortality Information System (*Sistema de Informação sobre Mortalidade* - SIM) available from the Department of Informatics of the Brazilian National Health System (DATASUS).

There was a decrease in the number of stroke-related deaths between 1997 and 2012 from 7.5% (95% CI: 6.7, 8.3) to 5.3% (95% CI: 4.5, 7.0) in men and from 7.1% (95% CI: 6.3; 7.9) to 5.1% (95% CI: 4.3; 5.9) in women (Table 1). The number of deaths was higher in the Southeast region in both men (N = 32,128) and women (N = 30,931), which corresponded to 51.2% (95% CI: 50.7, 51.7) and 49.8% (95% CI: 49.2, 50.4) of deaths, respectively (Table 1). The most common cause of death was intracerebral haemorrhagic stroke (I61) in men (N = 26,809, 42.7%) and women (N = 22,579, 36.4%), followed by non-specified ischaemic or haemorrhagic stroke (I64) in men (N = 25,000, 39.8%) and women (N = 21,939, 35.3%) (Table 1).

Overall, stroke deaths represented 3.8% (95% CI: 3.8; 3.8) of all deaths in this age group during the studied period. Proportional mortality due to stroke was greater in women (6.8%, 95% CI: 6.8; 6.9%) than in men (2.6%, 95% CI: 2.6; 2.7). In addition, the older age groups (45–50 years) presented higher proportional mortality in men (5.7%, 95% CI: 5.6, 5.8) and women (9.9%, 95% CI: 9.8; 10.0) than the younger age groups (Table 2).

**Table 2:** Proportional mortality* related to stroke** in Brazilian men and women aged 15 to 49 years between 1997 and 2012.

Characteristics	Proportional mortality^†^			
	Men		Women	
	%	95% CI	%	95% CI
**Age group (years)**				
15|–20	0.5	0.5; 0.6	1.7	1.6; 1.8
20|–25	0.6	0.6; 0.6	2.2	2.1; 2.3
25|–30	0.9	0.9; 0.9	3.0	2.9; 3.1
30|–35	1.6	1.6; 1.6	4.5	4.4; 4.6
35|–40	2.8	2.7; 2.9	7.0	6.9; 7.1
40|–45	4.3	4.2; 4.4	9.2	9.1; 9.3
45|–50	5.7	5.6; 5.8	9.9	9.8; 10.0
Regions				
North	2.4	2.3; 2.5	6.1	5.9; 6.3
Northeast	2.4	2.4; 2.4	6.8	6.7; 6.9
Southeast	2.9	2.9; 2.9	7.3	7.2; 7.4
South	2.5	2.4; 2.6	6.2	6.1; 6.3
Midwest	2.4	2.3; 2.5	6.3	6.1; 6.5
**Stroke subtypes**				
Subarachnoid haemorrhagic (I60)	0.4	0.4; 0.4	1,7	1.7; 1.7
Intracerebral haemorrhagic (I61)	1.1	1.1; 1.1	2.5	2.5; 2.5
Ischaemic (I63)	0.1	0.1; 0.1	0.2	0.2; 0.2
Non-specified (I64)^¥^	1.0	1.0; 1.0	2.4	2.4; 2.4
**Calendar year**				
1997	3.3	3.2; 3.4	8.3	8.1; 8.5
1998	3.4	3.3; 3.5	8.2	8.0; 8.4
1999	3.2	3.1; 3.3	8.2	8.0; 8.4
2000	3.0	2.9; 3.1	8.0	7.8; 8.2
2001	2.9	2.8; 3.0	7.6	7.4; 7.8
2002	2.7	2.6; 2.8	7.3	7.1; 7.5
2003	2.7	2.6; 2.8	7.5	7.3; 7.7
2004	2.7	2.6; 2.8	7.2	7.0; 7.4
2005	2.5	2.4; 2.6	6.7	6.5; 6.9
2006	2.6	2.5; 2.7	6.5	6.3; 6.7
2007	2.4	2.3; 2.5	6.4	6.2; 6.6
2008	2.4	2.3; 2.5	6.3	6.1; 6.5
2009	2.2	2.1; 2.3	5.6	5.4; 5.8
2010	2.2	2.1; 2.3	5.6	5.4; 5.8
2011	2.1	2.0; 2.2	5.6	5.4; 5.8
2012	2.1	2.0; 2.2	5.2	5.0; 5.4
Total	2.6	2.6; 2.7	6.8	6.8; 6.9

95% CI: 95% confidence interval.

*Proportion of all deaths among individuals aged 15 to 49 years between 1997 and 2012.

**International Statistical Classification of Diseases and Related Health Problems–10th revision (ICD-10) codes: I60, I61, I63 and I64^19,20^.

^†^In relation to the total number of deaths in people aged 15 to 49 years between 1997 and 2012, excluding ill-defined causes (R00–R99).

^¥^Stroke not specified as ischaemic or haemorrhagic.

Data source: Mortality Information System (*Sistema de Informação sobre Mortalidade* - SIM) available from the Department of Informatics of the Brazilian National Health System (DATASUS).

There was a decrease in proportional mortality from stroke from 3.3% (95% CI: 3.2; 3.4) in 1997 to 2.1% (95% CI: 2.0; 2.2) in 2012 in men and from 8.3% (95% CI: 8.1; 8.5) in 1997 to 5.2% (95% CI: 5.0; 5.4) in 2012 in women. Proportional mortality due to stroke was higher in the Southeast region for both men (2.9%, 95% CI: 2.9, 2.9) and women (7.3%, 95% CI: 7.2, 7.4). Proportional mortality was higher for the intracerebral stroke subtype (I61) in men (1.1%) and women (2.5%), followed by non-specified ischaemic and haemorrhagic stroke in men (1.0%) and women (2.4%). In addition, proportional mortality due to stroke was higher in women than in men for all age groups, regions, stroke subtypes, and years (Table 2).

**Figure 1:**
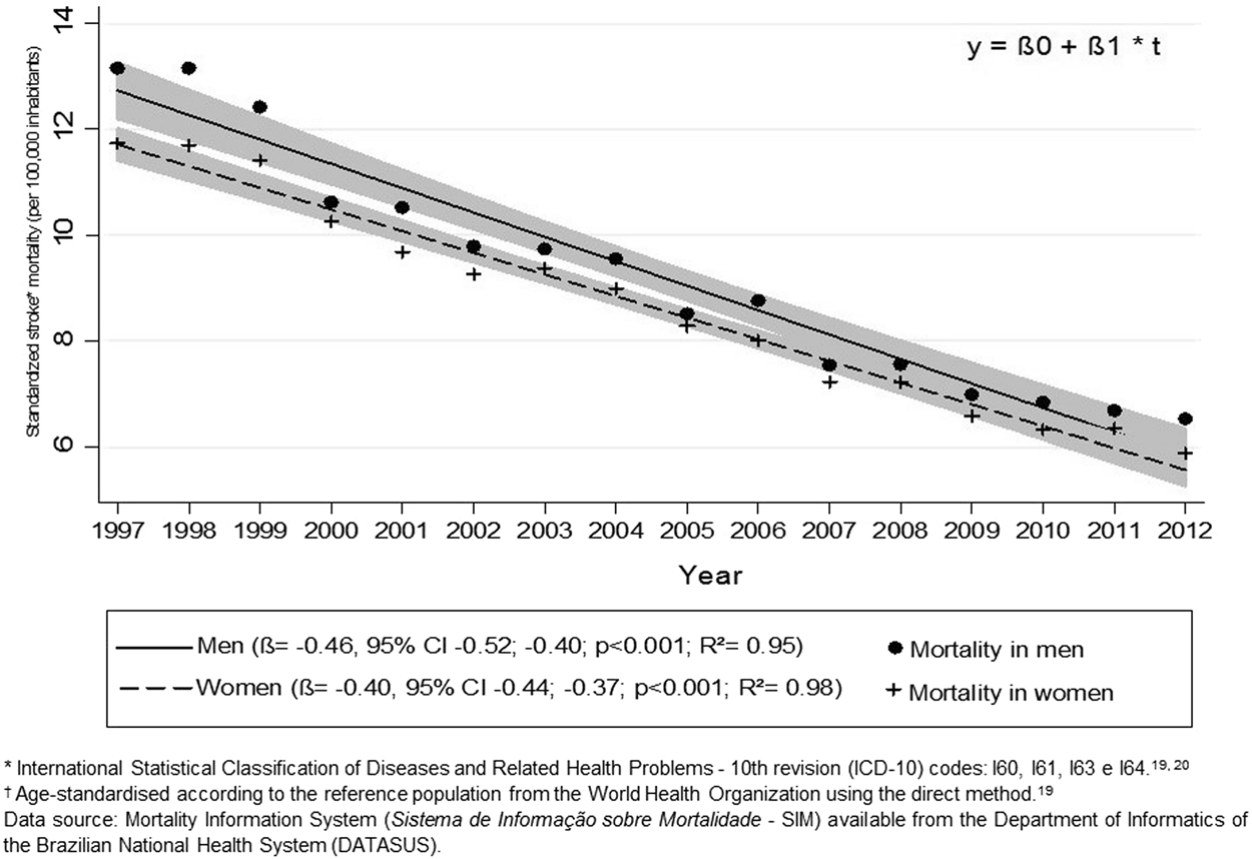
Proportional mortality related to stroke according to age† in Brazilian men and women.

There was a steady decline in stroke mortality rates between 1997 and 2012 for both men (β = −0.46, p < 0.001, R^2^ = 0.95) and women (β = −0.40, p < 0.001, R^2^ = 0.98) (Fig. 1), but non-significant interaction between sex and year (β = −0.051, p = 0.127, R^2^ = 0.96) (Table 3).

**Table 3:** Linear regression model with sex-years interaction of mortality related to stroke* in Brazilian men and women aged 15 to 49 years between 1997 and 2012.

Characteristics	β (95% CI)	p	R²
	y = β0 + β1* sex + β2* t + β3* sex* t		
**15|–50 years**			
Sex			
Men	*ref*	—	0.96
Women	−103.997 (−238.720; 30,726)	0.125	
Years	−0.461 (−0.508; −0.413)	<0.001	
Sex * Years	0.051 (−0.015; 0.118)	0.127	
**15|–20 years**			
Sex			
Men	*ref*	—	0.36
Women	19.471 (−25.333; 64.275)	0.381	
Years	−0.015 (−0.031; 0.0001)	0.052	
Sex * Years	−0.009 (−0.032; 0.012)	0.378	
**20|–30 years**			
Sex			
Men	*ref*	—	0.63
Women	57.869 (−13.343; 129.082)	0.107	
Years	−0.047 (−0.073; −0.022)	0.001	
Sex * Years	−0.028 (−0.064; 0.006)	0.107	
**30|–40 years**			
Sex			
Men	*ref*	—	0.92
Women	42.124 (−106.453; 190.701)	0.566	
Years	−0.339 (−0.391; −0.286)	<0.001	
Sex * Years	−0.021 (−0.095; 0.053)	0.565	
**40|–50 years**			
Sex			
Men	*ref*	—	0.96
Women	−480.346 (−847.377; −113.315)	0.012	
Years	−1.346 (−1.475; −1.216)	<0.001	
Sex * Years	0.238 (0.055; 0.421)	0.012	

95% CI: 95% confidence interval.

*International Statistical Classification of Diseases and Related Health Problems–10th revision (ICD-10) codes: I60, I61, I63 and I64^19,20^.

Data source: Mortality Information System (*Sistema de Informação sobre Mortalidade* - SIM) available from the Department of Informatics of the Brazilian National Health System (DATASUS).

**Figure 2:**
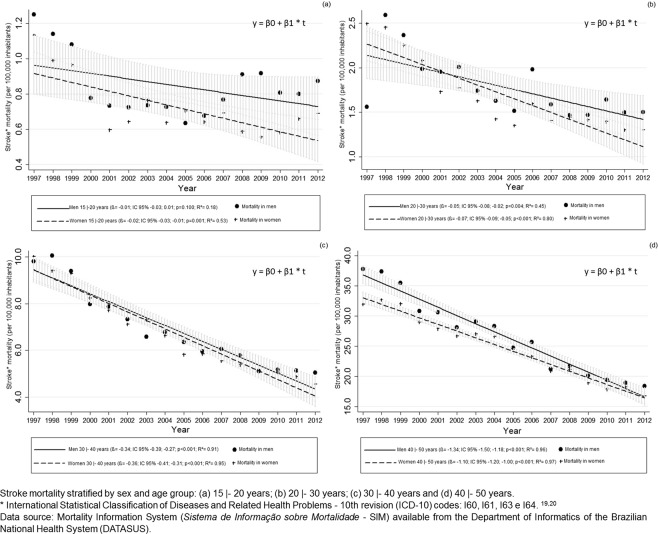
Time trends in stroke mortality according to sex and age group in Brazilian adults between 1997 and 2012.

Stroke mortality rates decreased for both sexes in Brazil throughout the study period in all age groups, but the difference was not statistically significant for men aged 15 to 19 years (Fig. 2). The decrease among men was higher in the 40- to 49-year-old age group during the first half of the analysis period, the period in which mortality rates among men were different than mortality rates among women (interaction between sex and year: β = 0.238, p = 0.012, R² = 0.96). For the other age groups 15 to 19 years (β = −0.009, p = 0.378, R² = 0.36), 20 to 29 years (β = −0.028, p = 0.107, R² = 0.63), and 30 to 39 years (β = −0.021, p = 0.565, R² = 0.92), there was non-significant interaction between sex and year (Table 3).

## Discussion

This present study analyzed the time trend of stroke among Brazilians aged 15 to 49 years between 1997 and 2012. Our main results showed: i) age-standardized stroke mortality decrease for men and women over the years, but non-significant interaction between sex and year was found; ii) the proportional mortality was higher among women than men; iii) there was a decrease in mortality for both sexes in all age groups, except by men aged 15 to 19 years; and iv) stroke mortality decrease was higher in men of 40–49-year-old age group during the first half of the study period.

Similar trends regarding stroke mortality in men and women were showed worldwide^25^ and in Brazil between 1979 and 2009^12^. We showed decrease in stroke mortality of 50.4% in men and 49.6% in women between 1997 and 2012 across Brazilian regions. Krishnamurthi *et al*.^26^ reported a 17.0% and 29.4% reduction in stroke mortality among young adults in developing and developed countries, respectively, between 1990 and 2013. Koton *et al*.^27^ found a decrease in stroke mortality in communities in the United States of 10.0 to 20.0% from 1987 to 2011, which was largely attributed to a decrease in rates among people under 65 years of age. In Brazil, Adami *et al*.^11^ found evidence of a 16% reduction in stroke mortality in Brazilians aged 15 to 49 years between 2008 and 2012. Stroke mortality decline in Brazilian population might be associated with improvements in both hypertension treatment and health care that target other risk factors for stroke in all socioeconomic groups^25,28^, in addition to advances in primary and secondary prevention, medical care, and efficacy of relevant medication^10^.

### Mortality by stroke subtypes

Higher number of deaths and a higher proportional mortality related to deaths were found due to haemorrhagic stroke of the intracerebral type. In individuals aged under 45 years, 50% of all stroke cases can be haemorrhagic stroke^29^. In low- and middle-income countries, such as Brazil^26^, the risk of haemorrhagic stroke death is higher than the risk of ischaemic stroke death. Greater death risk is related to different risk factors, aetiologies, prognoses, and treatment strategies for the most common subtypes of stroke (i.e. ischaemic and intracerebral haemorrhagic)^5,30^, and intracerebral haemorrhagic stroke is associated with the highest mortality rate. We could not analyse mortality rates by intracerebral haemorrhage, subarachnoid haemorrhage, and ischaemic stroke stratifications. More than one-third (37.59%) of the deaths recorded by DATASUS in Mortality Information System are referred to “Non-specified (NE) as ischaemic or haemorrhagic”^21^.

### Stroke mortality by sex and age

Previous studies have yielded divergent results as follows. Evidence show stroke mortality in young adults has no difference between sexes^31,32^. However, other study showed higher stroke mortality in men than in women^33^. Moreover, higher stroke has been found among older women^34,35^. Beyond sex differences, stroke mortality may vary according to the type of stroke, presence of comorbidities, severity, race/ethnicity, and geographic location^31,36^.

Our results showed higher proportional mortality due to stroke in women aged 15–49 years (6.8%) than in men (2.6%) in the same age group. This increase in proportional mortality in women is associated with specific risk factors for women^35^, e.g. pregnancy, perinatal period, or preeclampsia^37,38^, and the use of oral contraceptives^39^. Another possible explanation for sex differences in stroke mortality is the greater number of deaths due to external causes in men aged 15 to 49 years, which are the main cause of death for individuals in this age group (93,769 deaths in 2014)^21^, reducing the proportional mortality from other causes, including stroke.

Comparing each age group specifically, Fernandes *et al*.^13^ showed that stroke mortality in Brazil decreased in all assessed age groups with no differences between sexes. Controversely, no significant decrease in stroke mortality among men aged 15 to 19 years was found in this present study. Moreover, our data suggest non-linearity in the temporal trend in the 15–19 years age group in men and women. Data on stroke mortality for young adults are scarce, particularly for the youngest groups, which makes it difficult to compare our results with the previous literature. Global findings^40^ reported that between 1990 and 2013, the mortality rate for stroke in the 15–19-year-old age group is higher in men than women and the trend of reduction is similar between sexes with unknow reasons.

Our results showed a difference in the decrease in stroke between men and women only in the 40–49-year-old age group. Garritano *et al*.^41^ reported a continuous and linear reduction in the risk for stroke between 2000 and 2009 in Brazil in adults of both sexes between the ages of 30 and 49 years, with a higher reduction in men (32.38%) in the 40–50 age group when compared to women (32.06%). The accumulation of traditional and modifiable risk factors over time in men and the advanced age factor could explain this difference found in the upper age groups of this study^4^.

### Study Limitations

The present findings are important to improve understanding of stroke mortality time trends among young adults. However, some limitations should be highlighted: i) the coverage of Mortality Information System in the Northeast and North Brazilian regions is lower than that in other regions, which may have resulted in underreported cases, ii) incorrect register of ICD codes to classify stroke may underestimate found cases, i.e. misdiagnosed cases may have underestimated our findings, although the proportion of deaths due to ill-defined causes is approximately 7%^11,12,42^.

## Conclusion

The risk of death from stroke in men and women aged 15 to 49 years declined significantly in Brazil between 1997 and 2012 but did not significantly differ by sex, except by oldest age range group (40 to 49 years).
